# Editorial: Leptin Resistance in Metabolic Disorders: Possible Mechanisms and Treatments

**DOI:** 10.3389/fendo.2017.00300

**Published:** 2017-11-02

**Authors:** Toru Hosoi, Margherita Maffei

**Affiliations:** ^1^Department of Pharmacotherapy, Graduate School of Biomedical and Health Sciences, Hiroshima University, Hiroshima, Japan; ^2^CNR Institute of Clinical Physiology, Pisa, Italy; ^3^Obesity Center at Pisa University Hospital, Pisa, Italy

**Keywords:** leptin resistance, obesity, OBRb, age, sirtuin-1, sympathetic nervous system, endoplasmic reticulum stress, insulin

**Editorial on the Research Topic**

**Leptin Resistance in Metabolic Disorders: Possible Mechanisms and Treatments**

The steadily increasing prevalence of obesity in the last five decades is a serious global health threat, representing a major risk factor for diabetes, hypertension, and hyperlipidemia, i.e., metabolic syndrome. A seminal finding to the understanding of the mechanisms leading to body weight dysregulation and the onset of obesity was the discovery of leptin, from the Greek term *leptos* (thin), identified by Friedman and collaborators at The Rockefeller University in 1994 (1). Leptin exerts its anti-obesity action by inhibiting food intake and inducing energy expenditure (2). This effect was demonstrated in leptin-deficient individuals and raised expectations regarding its potential as a drug to reduce body weight in obese patients (3). However, obese patients and mice display high levels of circulating leptin (4) and do not respond to leptin treatment, a condition known as leptin resistance (5) with underlying mechanisms that have not yet been fully elucidated (6).

This study presents five review articles which discuss the latest findings on the molecular and cellular mechanisms that underlie leptin resistance and which propose potential therapeutic strategies to overcome this condition.

Roujeau et al. paper is focused on leptin receptor, which is present in several splicing isoforms, including OBRa, OBRb, OBRc, OBRd, and OBRf. Among them, OBRb is the longest isoform, and to date, the only one demonstrated to actively transduce the leptin signal. The authors emphasize the importance of establishing screening systems to identify molecules able to activate OBRb and thus improve leptin sensitivity. One proposed screening strategy is a high-throughput assay system to identify candidate drugs/peptides molecules based on the recently developed homogenous time-resolved fluorescence technology (7). In addition, the authors discuss several possible mechanisms of leptin resistance, including decreased leptin transport into the brain, over-activation of the negative feedback regulator of leptin signaling, impaired leptin receptor trafficking, and endoplasmic reticulum (ER) stress.

The middle-aged population is prone to weight gain and obesity exacerbates the risk of developing age-associated diseases. The mechanisms underlying age-associated weight gain have yet to be completely elucidated and a more comprehensive understanding of this subject is key to the prevention of obesity in the elderly. On this topic, Sasaki discuss age-associated weight gain, focusing on leptin and sirtuin-1 (SIRT1), a nicotinamide adenine dinucleotide-dependent protein deacetylase. SIRT1 is involved in promoting longevity through inhibiting caloric intake. SIRT1 has also been implicated in the reduction of leptin resistance through suppression of food intake. Sasaki discuss a possible strategy for treating aging- and diet-induced obesity through modulating hypothalamic SIRT1 function and nicotinamide adenine dinucleotide concentration.

Through sympathetic nervous system activation, leptin induces the thermogenesis of brown adipose tissue and lipolysis of white adipose tissue (WAT). Sympathetic neuro-adipose junctions have been implicated as key determinants of WAT tissue lipolysis (8), but what remains to be clarified is whether and how leptin peripheral resistance may affect these junctions and their capacity to transmit sympathetic nervous system signals. Within their contribution, Barateiro et al. discuss leptin resistance in the context of neuro-adipose connection, lipolysis, and thermogenesis.

Increasing evidence points to the central role of insulin as an important determinant of metabolism regulation. Of note, neuronal populations targeted by insulin and leptin are partly overlapping in the hypothalamic regions that regulate energy homeostasis and feeding behavior. Furthermore, the two hormones share intracellular pathways and exert a reciprocal interaction in the central nervous system to regulate metabolism and finely modulate appetite and satiety. Insulin and leptin resistance are often associated in obesity (9). Thon et al. describe the crosstalk between leptin and insulin action in the CNS to regulate energy homeostasis.

Obesity is associated with ER stress (10) and recent evidence points to this stress as a contributor to the development of leptin resistance. Therefore, targeting ER stress may be one strategy for the treatment of obesity caused by leptin resistance. Hosoi and Ozawa’s contribution deals with the mechanisms of ER stress-induced leptin resistance and a possible pharmacological strategy to improve leptin sensitivity by targeting ER stress.

By gathering contributions from different research areas, this issue provides a rich perspective on the molecular mechanisms triggering leptin resistance that, as we learn from these papers, may originate from causes ranging from systemic to molecular derangements. Since 1994, leptin research has provided knowledge on the mechanisms regulating energy homeostasis and on the relative dysfunctions leading to obesity. In the past 23 years, we have discovered that leptin lies in the center of a highly complex network that we are only starting to elucidate; a progressive understanding of how the system works will hopefully lead to the development of therapeutic (and not only) pharmacological strategies to prevent obesity.

## Author Contributions

All authors listed have made a substantial, direct, and intellectual contribution to the work and approved it for publication.

## Conflict of Interest Statement

The authors declare that the research was conducted in the absence of any commercial or financial relationships that could be considered as a potential conflict of interest.
